# Mevinphos: MAK Value Documentation, addendum – Translation of the German version from 2024

**DOI:** 10.34865/mb778634e11_2ad

**Published:** 2026-06-30

**Authors:** Andrea Hartwig, MAK Commission

**Affiliations:** 1 Institute of Applied Biosciences, Department of Food Chemistry and Toxicology, Karlsruhe Institute of Technology (KIT) Adenauerring 20a, Building 50.41 76131 Karlsruhe Germany; 2 Permanent Senate Commission for the Investigation of Health Hazards of Chemical Compounds in the Work Area, Deutsche Forschungsgemeinschaft, Kennedyallee 40, 53175 Bonn, Germany. Further information: Permanent Senate Commission for the Investigation of Health Hazards of Chemical Compounds in the Work Area | DFG

**Keywords:** mevinphos, insecticide, pesticide, toxicity, evaluation, acetylcholinesterase inhibitor

## Abstract

Mevinphos [7786-34-7] is not approved in the European Union as an insecticide. The previous MAK Value documentation and addendum do not reflect the current data situ­ation of the substance. The MAK Commissiondecided that a new evaluation is not of high priority. The MAK value and the other classifications are therefore suspended and the substance is listed in the Section II c of the List of MAK and BAT Values for substances no longer evaluated.

**Table:** 

**MAK value**	**see Section II c of the List of MAK and BAT Values**
**Peak limitation**	**–**
	
**Absorption through the skin**	**–**
**Sensitization**	**–**
**Carcinogenicity**	**–**
**Prenatal toxicity**	**–**
**Germ cell mutagenicity**	**–**
	
**BLW (2023)**	**reduction of the acetylcholinesterase activity in erythrocytes to 70% of the reference value^a)^**
	
Synonyms	(2-methoxycarbamoyl-1-methylvinyl)dimethylphosphate phosdrin
Chemical name (IUPAC)	methyl (E)-3-dimethoxyphosphoryloxybut-2-enoate
CAS number	7786-34-7
Molar mass	224.15 g/mol
Melting point	cis: 6.9 °C (IFA 2023) trans: 21 °C (IFA 2023)
Boiling point	decomposes when heated (IFA 2023)
Density at 20 °C	1.25 g/cm^3^ (IFA 2023)
Vapour pressure	< 0.001 hPa (IFA 2023)
log K_OW_	1.2 (IFA 2023)
Solubility	completely miscible with water (IFA 2023)
**1 ml/m^3^ (ppm) **≙** 9.301 mg/m^3^**	**1 mg/m^3^ **≙** 0.108 ml/m^3^ (ppm)**

^a)^ The BLW (biological guidance value) is derived as the ceiling value because of acute toxic effects.

Note: The substance can occur simultaneously as vapour and aerosol.

This addendum was prepared because the previous evaluations no longer reflect the data currently available for the MAK value and for the designations and classifications of the substance. Mevinphos is used as an insecticide. It is an organophosphate that acts by inhibiting cholinesterase in the blood and tissues without the need for activation (Greim 2002, available in German only). The biological guidance value (BLW) for acetylcholinesterase inhibitors (reduction of the acetylcholinesterase activity in erythrocytes to 70% of the reference value; Lewalter 1995; Weistenhöfer et al. 2024) therefore applies to mevinphos. The BLW is derived as the ceiling value because of acute toxic effects. However, it was not investigated whether this is the most sensitive end point.

In 1961, a MAK value of 0.01 ml/m^3^ (0.093 mg/m^3^) was set and the substance was designated with an “H” (for substances which can be absorbed through the skin in toxicologically relevant amounts) (Henschler 1972, available in German only). In the addendum from 2002, mevinphos was assigned to Peak Limitation Category II with an excursion factor of 2 (Greim 2002).

In the European Union, the use of mevinphos as an insecticide is not permitted under Regulation (EC) 1107/2009 concerning the placing of plant protection products on the market (European Commission 2022; European Parliament and European Council 2009). In the Federal Republic of Germany, mevinphos was approved for use as a plant protection product from 1971 to 1994, in the former German Democratic Republic it could be used until 1994 (BVL 2010).

The previous evaluations (MAK value documentation and addendum) do not reflect the currently available data. However, a re-evaluation of the substance is not a priority. Therefore, the MAK value, the peak limitation and the “H” designation have been withdrawn and mevinphos has been allocated to Section II c of the List of MAK and BAT Values (DFG 2022). This section lists substances for which the previous MAK values, designations and classifications have been withdrawn and which are no longer being reviewed at present.
